# Molecular Dynamics Simulation of the Thermomechanical and Tribological Properties of Graphene-Reinforced Natural Rubber Nanocomposites

**DOI:** 10.3390/polym14235056

**Published:** 2022-11-22

**Authors:** Zepeng Wang, Minglong Su, Xinwu Duan, Xiulong Yao, Xiaoying Han, Junping Song, Lianxiang Ma

**Affiliations:** College of Electromechanical Engineering, Qingdao University of Science and Technology, Qingdao 266061, China

**Keywords:** molecular dynamics simulation, nanocomposites, thermodynamic properties, tribological properties

## Abstract

The thermomechanical and tribological properties of graphene (GNS)-reinforced NR were investigated using molecular dynamics (MD) simulations. The amorphous molecular dynamics models of two nanocomposites, i.e., natural rubber (pure NR) and graphene/natural rubber (GNS/NR), were established. In addition, the thermodynamic properties of the two materials, before and after the incorporation of graphene into the natural rubber matrix, were investigated through analytical comparison. The results showed that after the graphene was added to the rubber matrix as a reinforcing material, the elastic modulus and shear modulus were increased by 110% and 94.8%, respectively, the tensile property was increased by 178%, the overall thermal conductivity of the composite system was increased by 59%, the glass transition temperature increased from 223 K to 236 K, and the rigidity of the material matrix was significantly improved. The inherent interactions and wear mechanisms of the polymer nanocomposites were discussed at the atomic scale by analyzing the changes in temperature, atomic velocity, relative atomic concentration, and radial distribution functions at the friction interface in the thickness direction.

## 1. Introduction

Polymer composites have become one of the most promising materials in the field of materials science today due to their excellent mechanical, thermal, electrical, and chemical properties [1,2], and they are widely used in production and industrial engineering [3]. It is therefore necessary to deeply understand their ontological properties in terms of their physical microenvironment and chemical structure [4,5], especially their thermomechanical and tribological properties.

With the rapid development of polymer nanocomposites, molecular dynamics (MD) simulation has become an effective alternative method for studying and predicting material properties at the microscopic atomic scale, providing microscopic information and mechanisms of molecular interactions. Simultaneously, MD simulation can also be used to analyze the experimental results. Therefore, molecular dynamics simulation studies are essential to evaluate and study the thermodynamic properties of polymer nanocomposites. M. Ding et al. [6] calculated the thermomechanical properties of cross-linked epoxy resin/functionalized carbon nanotube composites based on molecular dynamics simulations, and the simulation results showed that the overall thermal conductivity of epoxy nanocomposites doped with carbon nanotubes were all improved, and the doping of carbon nanotubes led to the improvement in the glass transition temperature and mechanical properties of the composite system. Y. Li et al. [7,8,9,10,11] investigated the mechanical and frictional wear properties of carbon nanotube/NBR (nitrile-butadiene rubber) composites using molecular dynamics simulations. The results showed that the introduction of CNTs reduced the friction coefficient of the composites by about 38% and the average wear rate by about 60% under different normal loads. In addition, the tribological enhancement of CNTs/polymer composites was further investigated. Q, Xue et al. [12] used CNTs as a reinforcing agent and demonstrated that they could inhibit the adhesion and wear of the PI matrix, thus reducing the friction coefficient and frictional wear volume. H. Chen et al. [13] simulated the CNT pull-out process and a series of friction processes between the composite and metal substrate by MD simulation to explain the motion state between the atoms of each part from the energy perspective, studying the force on the CNT and the change in the displacement of each part to infer the motion attitude. K Pan et al. [14] simulated the detachment process of cross-linked polydimethylsiloxane (PDMS) from functionalized graphene (fG) using the molecular dynamics simulation method using ReaxFF potential function. The effects of graphene modified by three different functional groups—hydroxyl (-OH), amino (-NH2), and carbon groups (-CH3)—on the interfacial mechanical properties of the composites were investigated. The results showed that different functional groups had a significant influence on the interfacial mechanical properties of the composites.

In this study, the effects of hydrogenated graphene on the thermomechanical and tribological properties of natural rubber were investigated. Firstly, a pure natural rubber (polyisoprene pure rubber) model and hydrogenated graphene/natural rubber (GNS/NR) composite model were constructed using Materials Studio [15,16,17,18] software to simulate and calculate the tensile properties, shear modulus, glass transition temperature, and thermal conductivity of the materials. Next, a molecular model of the metallic iron atomic layer and the polymer matrix was developed to simulate the frictional wear behavior. Finally, by analyzing the microscopic information, such as atomic concentration, atomic velocity distribution, and radial distribution function at the friction interface, the microscopic mechanism of the thermodynamic and frictional wear properties of graphene-enhanced natural rubber was explored and revealed at the microscopic level. This study overcomes the limitations of previous frictional wear macroscopic tests and provides a new method and theoretical guidance for the study of inorganic nanomaterial-reinforced polymer composites.

## 2. Materials and Methods

### 2.1. Model Construction

**Figure 1:**
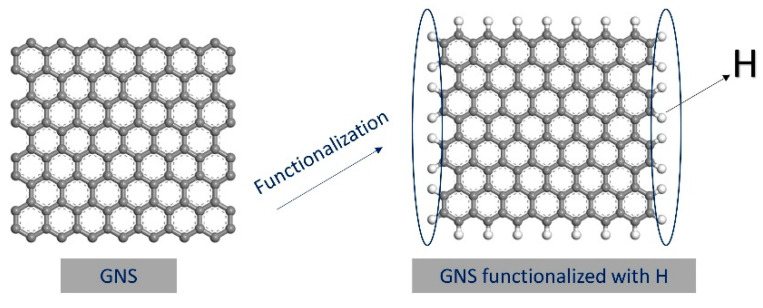
Graphene functionalized with hydrogen atoms.

In this study, a nanocomposite model system with periodic boundary conditions was developed using Materials Studio (8.0). The “Amorphous Cell Package” and “Forcite” modules in Materials Studio were used for modeling and simulation. The COMPASS II force field [19], which can provide interatomic interactions and qualitatively describe the physical and mechanical properties [20], as well as the thermodynamic properties of the polymer, was used in the simulations. The first step was to establish a periodic cubic lattice with the dimensions of 45 Å × 45 Å × 45 Å and to construct a molecular chain of polyisoprene (C5H8)_n_ natural rubber with a repeating unit of a polymerization degree of 30. Then, a monolayer graphene GNS with the dimensions of 11.30 Å × 12.30 Å was constructed, in which the edge of GNS was functionalized with hydrogen atoms, and subsequently, the hydrogenated monolayer graphene was placed at the center of the lattice, as shown in Figure 1.

**Figure 2:**
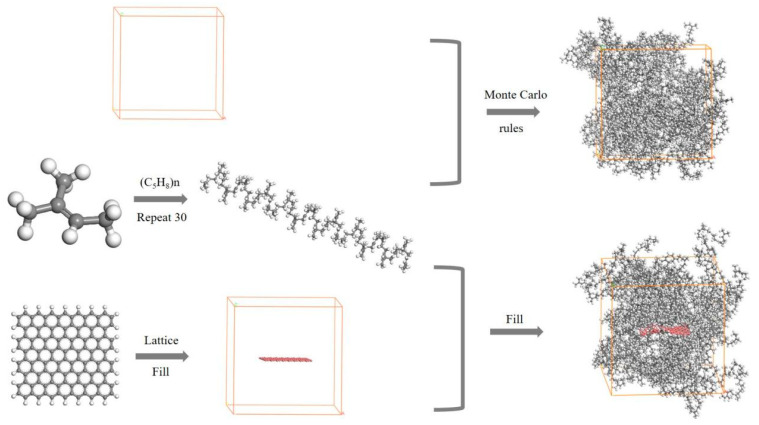
Molecular model: NR amorphous molecular model and GNS/NR amorphous molecular model.

Eventually, the polyisoprene molecular chains were gradually and randomly filled into the lattice, according to Monte Carlo rules, until the lattice density reached the theoretical value of 0.92 g/cm^3^. For the Pure NR amorphous molecular model, the molecular model was constructed in the same way, with a preset density of 0.92 g/cm^3^. The resulting model is shown in Figure 2.

### 2.2. Model Optimization

Since the internal energy of the constructed molecular models was too high and the molecules were in a highly unstable state, the conjugate gradient method was used to calculate the total potential energy of the two amorphous molecular models in a geometrically optimized manner to achieve the minimal local energy configuration of the molecular system. The energy convergence accuracy reached the root mean square value of 0.00001 kcal/mol, and the force convergence accuracy reached 0.0001 kcal/mol/Å to obtain the global minimum energy configuration. In order to improve the accuracy of the simulation result, the model was further equilibrated by performing five annealing cycles of simulation in the temperature range of 150 K to 350 K. It was required to obtain the molecular dynamics equilibrium of the two molecular conformation systems at the reference temperature of 150 K, to set the temperature growth step to 50 K, and to run a constant temperature isotropic (NVT system synthesis) system for 50 ps at the target temperature. After the simulation of several annealing cycles at high and low temperatures, the structure of the molecular system was further relaxed, and the model configuration was gradually rationalized to a stable state with minimal local energy. Finally, the two systems were subjected to an isothermal isobaric (NPT system synthesis) process of molecular dynamics equilibrium at room temperature 298 K, pressure 101 KPa, and time step 1 ps. The algorithm of Nose and Berendsen was used to control the temperature and pressure during the simulation. When the energy and density of the entire system do not change significantly with time, we believe that the molecular model has reached the most stable state, with minimal energy. The final densities of the pure NR model and the GNS/NR model were 0.897 g/cm^3^ and 0.959 g/cm^3^, respectively, which were consistent with the true density of natural rubber polyisoprene of 0.92 g/cm^3^.

### 2.3. Thermodynamic and Tribological Simulation

The mechanical properties of both the pure NR and GNS/NR composites were calculated by the method of constant strain technique to calculate the stress–strain properties and shear modulus of the materials. The glass transition temperature is determined by calculating the specific volumes of the two materials at different temperatures, obtained by linearly fitting the resulting data and comparing them. Based on Fourier’s law, non-equilibrium molecular dynamics simulations were used to calculate the thermal conductivity of the two materials separately.

In order to simulate the tribological properties of the pure NR and GNS/NR composites, two three-layer tribological sub-models were established. Combined with the actual working conditions of rubber tires, the iron atom layer was selected for the upper and lower layers of the friction sub-model, and the iron atom layer size was 45 Å × 45 Å × 11.5 Å. Pure NR and GNS/NR were used as intermediate layers to fix the upper and lower iron atomic layers, and the two friction sub-models were optimized separately using the above-mentioned method of optimizing the amorphous model, and the fixation of the metal atomic layer was removed after the optimization was completed. To obtain the tribological properties of the polymer composite layer and the metal layer, a positive pressure of 1.01 × 10^−4^ GPa and a relative sliding friction velocity of 0.1 Å/ps were given to the upper iron atomic layer, the temperature was set to 298 K in an ambient environment, and the whole system was run for 600 ps under the NVT system synthesis to obtain the trajectory file of the frictional wear situation over time, which was used to analyze the material frictional wear properties.

## 3. Results and Discussions

### 3.1. Thermodynamic Property

#### 3.1.1. Tensile Properties Simulation

**Figure 3:**
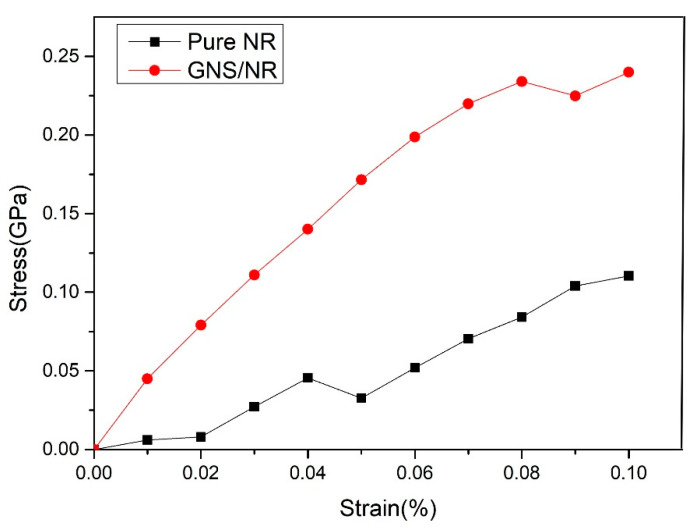
Stress-strain curves of the pure NR and GNS/NR composites.

The tensile properties of the pure natural rubber and graphene/natural rubber nanocomposites were simulated using the constant strain method [21,22]. The tensile properties of the pure NR and GNS/NR composites can be calculated through the uniaxial stretching process in the X-direction to obtain the stress–strain curves for both materials, as shown in Figure 3.

It can be seen from Figure 3 that in the strain range of 0 to 0.08, the tensile stress of the matrix of the two material showed an increasing trend, and the tensile stress of the GNS/NR composite matrix increased to 0.24 GPa in a linear manner. By comparing these results with literature data [23], this stress–strain behavior was within a reasonable range of variation, and the accuracy of the calculation method for the tensile behavior using molecular dynamics simulations was verified.

The points with large errors were eliminated, and the slope of the fitted stress–strain curve was used to calculate the modulus of elasticity. The modulus of elasticity of the two material substrates, pure NR and GNS/NR, was 1.150 GPa and 2.416 GPa, respectively. When hydrogenated GNS was added to the material as a reinforcement system, the modulus of elasticity of the NR substrate was increased by 110%, and the tensile properties were improved by 178% (at the strain of 0.08). This phenomenon is due to the adsorption of VDW and electrostatic forces on the surface of GNS. A spatially reinforcing interface was formed between GNS and the natural rubber nanopolymer matrix, which made the polymer chains adsorbed tightly around the GNS. When the whole system was subjected to external forces, the adsorption of GNS on the polymer matrix limited the shedding of polymer chains within the matrix. Consequently, when the GNS/NR material matrix was subjected to an external stretching force, a larger stretching force was needed to achieve the same strain; thus, the material matrix had an increased elastic modulus. The high elastic modulus prevented cracking during stretching and further improved the tear strength of the matrix, thus extending the service life of the rubber composite matrix.

**Figure 4:**
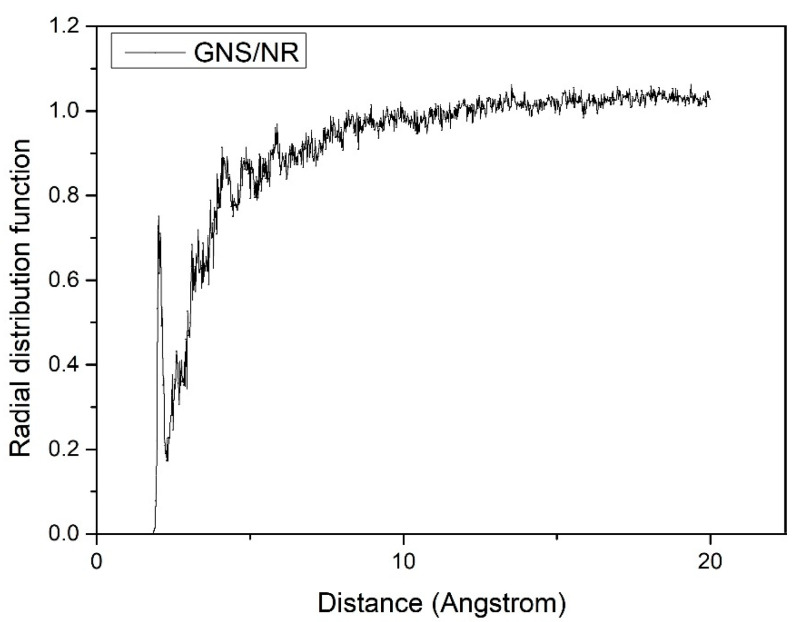
Radial distribution function between graphene and the natural rubber matrix during the tensile process.

In order to explore this reaction more deeply, the mechanism was verified and analyzed. The radial distribution function between GNS and the natural rubber polymer matrix during the stretching process was calculated and extracted for analysis. As shown in Figure 4, after adding GNS to the rubber material matrix as a reinforcing material, when the contact distance between atoms in the Z direction was greater than 10 Å during the stretching process, the RDF values of the radial distribution function between the GNS and the atoms within the polymer matrix tended to be in dynamic equilibrium. The unique two-dimensional lamellar structure of GNS, with a higher specific surface area, could adsorb and adhere to more of the polymer matrix. When the composite matrix was subjected to tensile forces, the VDW forces, as well as the electrostatic adsorption between the upper and lower surfaces of the GNS and the polymer matrix, prevented the polymer chains from moving in the tensile direction. Therefore, higher tensile stress is required to obtain the same tensile strain as that of pure NR, which shows the better mechanical properties of the composite.

#### 3.1.2. Shear Modulus and Bulk Modulus

To further investigate the effect of graphene on the mechanical properties of rubber nanomaterials, the bulk modulus (*K*) and the shear modulus (*G*) of both the pure NR and GNS/NR were calculated based on the optimized final structure. The constant strain approach was employed by exerting 20 in 6 directions (i.e., x, y, z, yz, xz, xy) of the systems. Finally, the 6*6 stiffness matrices (*C_ij_*) and the compliance matrices (*S_ij_*) were obtained using the following equations:

(1) $$C_{ij}=\sigma_{i}$$

(2) $$S_{ij}=\varepsilon_{j}/\sigma_{i}$$

The upper limits of *K* and *G* are determined by the following the Voigt expressions:

(3) $$K_{voigt}=(C_{11}+C_{22}+C_{33}+2C_{12}+2C_{13}+2C_{23})/9$$

(4) $$G_{voigt}=(C_{11}+C_{22}+C_{33}+3C_{44}+3C_{55}+3C_{66}-C_{12}-C_{13}-C_{23})/15$$

The lower limits of *K* and *G* are determined by the following the Reuss expressions:

(5) $$K_{Reuss}=1/(S_{11}+S_{22}+S_{33}+2S_{12}+2S_{13}+2S_{23})$$

(6) $$G_{Reuss}=15/\left[4(S_{11}+S_{22}+S_{33})-4(S_{12}+S_{13}+S_{23})+3(S_{44}+S_{55}+S_{66})\right]$$

Finally, the actual values of *K* and *G* are determined by the Hill theory, averaging the corresponding values of *K* and *G* obtained from the Voigt and Reuss theories. The corresponding equations are given as follows:

(7) $$K_{Hill}=(K_{voigt}+K_{Reuss})/2$$

(8) $$G_{Hill}=(G_{voigt}+G_{Reuss})/2$$

As can be seen from Table 1, the shear moduli of the two materials, pure NR and GNS/NR, were calculated by simulation to be 1.441 GPa and 2.807 GPa, respectively. The analysis of the data results showed that when GNS was added to the natural rubber matrix as a reinforcing material, the shear modulus and bulk modulus of the composite matrix were increased by 94.8% and 110.1%, respectively. Due to the van der Waals force and the electrostatic force adsorption between GNS and the polymer matrix, the polymer chains were tightly adsorbed on the GNS surface. When the entire matrix system was subjected to the external shear force, GNS/NR required a greater shear force than the pure NR matrix to achieve the same shear strain. The addition of graphene significantly improves the shear properties and resistance to volume change of the rubber material matrix, thereby extending the service life of the rubber.

#### 3.1.3. Simulation of Glass Transition Temperature

**Figure 5:**
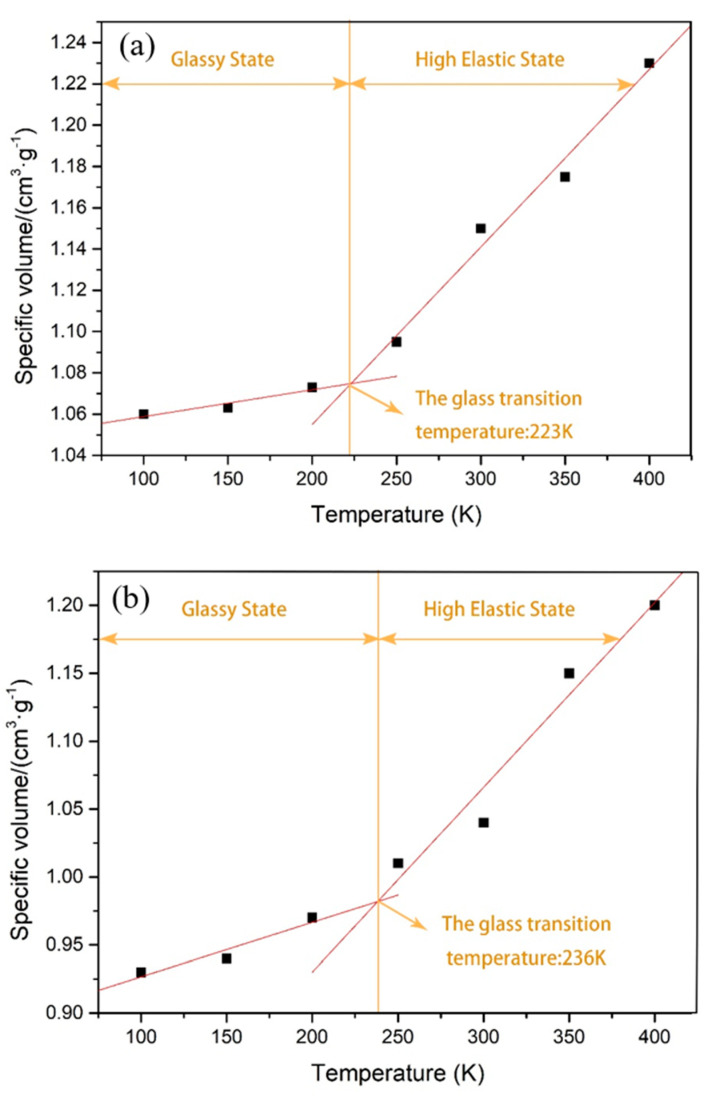
(**a**) Transition temperature curve for NR glass; (**b**) transition temperature curve for GNS/NR glass.

The glass transition temperature serves as the polymer’s lower service temperature limit and reflects the material’s low temperature resistance. As seen in Figure 5, there are significant discontinuities in the slope of the specific volume against temperature for the material, indicating that the rubber experiences a secondary phase transition from the glassy to the rubbery state at these discontinuities. To determine the precise glass transition temperature, a segmented linear regression was applied to all data points. The graph demonstrates that the material’s specific volume increases gradually as the temperature increases, then increases quickly at the junction of the two fitted lines, showing that the rubber–glass transition occurs at the intersection point and that the temperature corresponding to the intersection point is the rubber–glass transition temperature. 

As demonstrated in Figure 5, the addition of GNS to the natural rubber matrix caused the glass transition temperature to rise by just 5.8%, from 223 K to 236 K. This suggests that the addition of GNS to the natural rubber nanocomposite matrix increases the stiffness, restricts the movement, and decreases the flexibility of the rubber molecular chain. It also increases the hardness and the glass transition temperature of the rubber, to some extent, increasing the glass transition temperature and decreasing the low temperature resistance of the rubber. However, compared to the enhancement of the rubber’s mechanical qualities and tribological capabilities, the low-temperature resistance is within an acceptable range.

#### 3.1.4. Thermal Conductivity Simulation

The non-equilibrium molecular dynamics simulation method, based on Fourier’s law, was used to calculate the thermal conductivity of the two materials. The construction and optimization processes of both polymer materials were consistent with the above results. The time step was set to 1 fs, and the exchange time step was 250 at 298 K. The Berendsen thermostat was selected for temperature control, and the entire process was simulated using the COMPASS force field. The simulation results are shown in Table 2.

The addition of GNS increased the thermal conductivity of the natural rubber material matrix by 59%, as shown in Table 2. This is due to the fact that the addition of GNS to the interior of the natural rubber matrix increased the molecular chain orientation and the ordered structure led to an increase in the average free range of phonon diffusion and a weakening of phonon scattering, thereby enhancing the thermal conductivity of the entire rubber matrix system.

### 3.2. Tribological Performance Simulation

**Figure 6:**
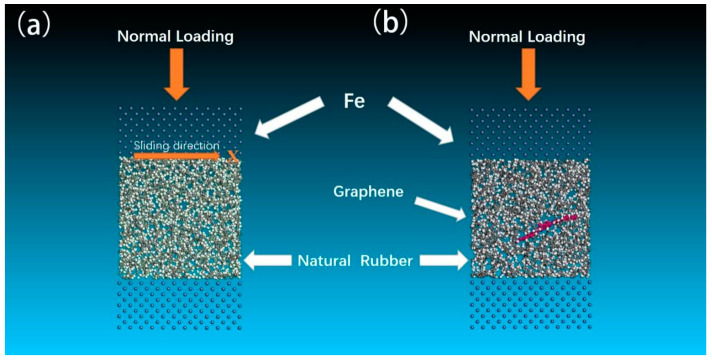
Molecular friction pair configuration: (**a**) pure natural rubber and iron layer model; (**b**) graphene/natural rubber and iron layer model.

In order to study the tribological performance of the two materials, i.e., pure NR and GNS/NR, two three-layer tribological sub-models were constructed, as shown in Figure 6. Combined with the working conditions in the actual application, iron atoms were chosen as the base and top layers of the models, and a polymer model was used as the middle layer. The dimensions of both the base and top iron atom layers were 45 Å × 45 Å × 11.5 Å. The upper and lower iron atomic layers were fixed, and the two friction sub-models were optimized using the above optimization method for two amorphous molecular models. The immobilization of the upper and lower iron atomic layers was eliminated after a series of optimizations. To obtain the relative sliding tribological properties of the metallic iron atomic layer and the polymer composite configuration, a normal positive pressure load of 101 KPa was applied to the top layer, which was run for 400 ps at 0.1 Å/ps under the NVT system synthesis with a temperature of 298 K and a pressure of 101 KPa. The trajectory of the atoms in the sliding direction and the forces between the atoms were obtained and used to analyze the tribological properties.

**Figure 7:**
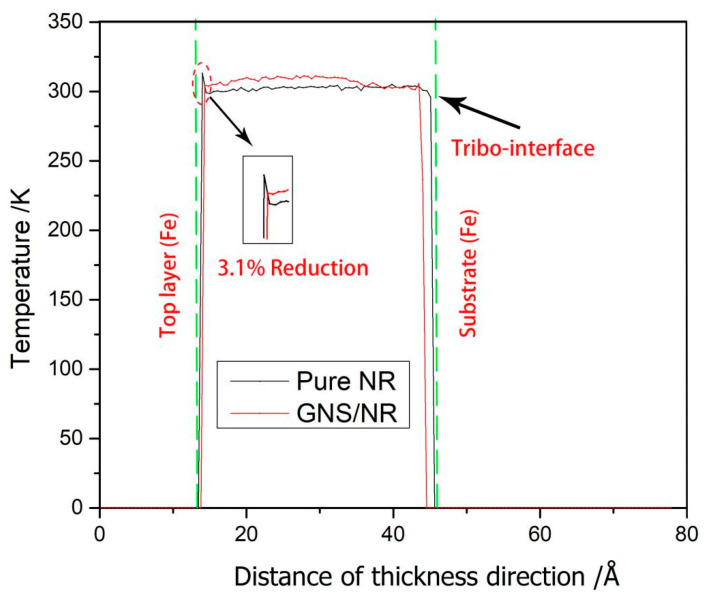
Temperature distribution of the pure NR and GNS/NR composites in the thickness direction.

**Figure 8:**
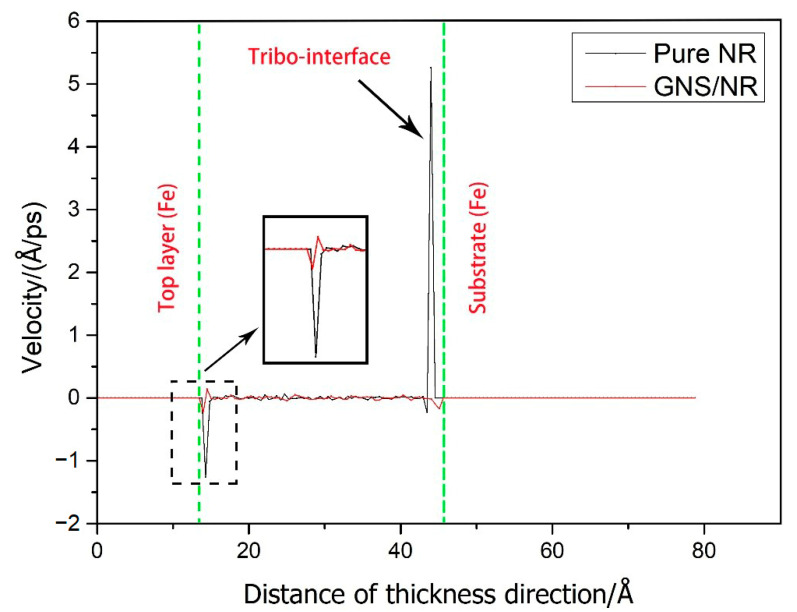
Velocity distribution of pure NR and GNS/NR composites in the thickness direction.

To investigate the mechanism affecting the tribological properties of both the pure NR and GNS/NR nanocomposites, the profiles of atomic temperature and velocity along the thickness direction during the molecular dynamics friction simulation were calculated and extracted. The results are shown in Figure 7 and Figure 8.

From Figure 7, for Pure NR (polyisoprene), around the top friction contact region, the temperature reached 313 K inside the material model along the thickness direction (at about 14 Å in the Z-direction); for the GNS/NR composite reinforced with GNS, the temperature was reduced by 3.1%. It has been shown that the more atoms around the friction interface between the top layer of the Fe metal and the polymer, the greater the energy dissipation. Based on Fleisher’s tribological theory [24], energy dissipation occurs during the friction process, and frictional work is eventually converted into heat, leading to an increase in the temperature of the whole system. The addition of GNS reduced the interaction between the polymer molecules and the metal atoms, which lowered the coefficient of friction and eventually lowered the temperature increase generated by the entire system.

It can be further seen from Figure 8 that the pure NR substrate showed a velocity peak of 5.26 Å/ps at the friction interface, with faster molecular motion and stronger interaction with the metal Fe layer. The GNS/NR composite, on the other hand, did not show a significant velocity peak. The reason for this result is that the addition of GNS to the NR matrix increases the stability of the interface and improves the wear resistance of the composite due to the adsorption of electrostatic forces on the GNS surface and the van der Waals forces between the molecular chains and polyisoprene. As a result, the interaction with the metal Fe atomic layer was reduced, and the intermolecular motion was limited.

**Figure 9:**
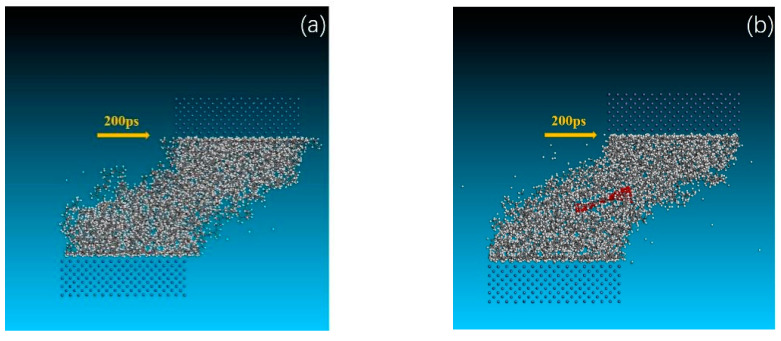

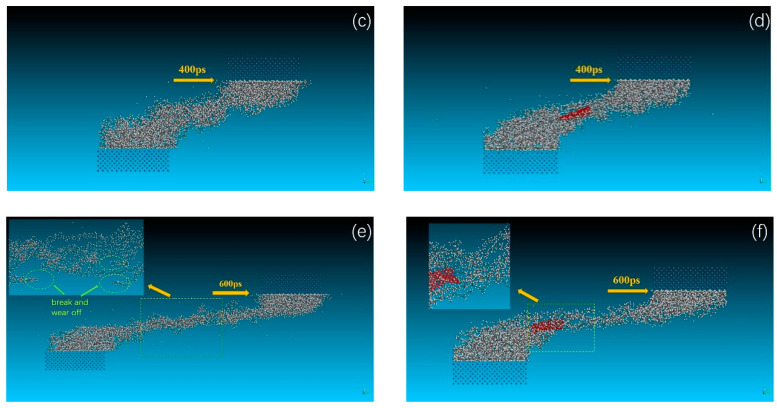
Friction process of the pure NR (**a**,**c**,**e**) and the GNS/NR composite (**b**,**d**,**f**) with a Fe layer at 200 ps, 400 ps, and 600 ps.

To obtain more insight into the wear mechanism of the two materials analyzed, Figure 9 shows snapshots of the different stages of the friction process (200 ps, 400 ps, 600 ps). From Figure 9, it is clear that pure NR, with weak mechanical properties, experiences greater deformation under shear (Figure 9a,c), fractures (Figure 9e) and finally wears away from the NR substrate. The number of atoms fractured, as shown in Figure 9e, can be identified as the wear molecule of the friction process, and the wear rate of pure NR is 6.35% under the same conditions, while the GNS/NR composite is observed to have no obvious wear phenomenon in the final state under the same conditions, and the deformation of the material can only be observed under shear (Figure 9b,d,f), further confirming that GNS has an enhancement effect on NR material wear. 

**Figure 10:**
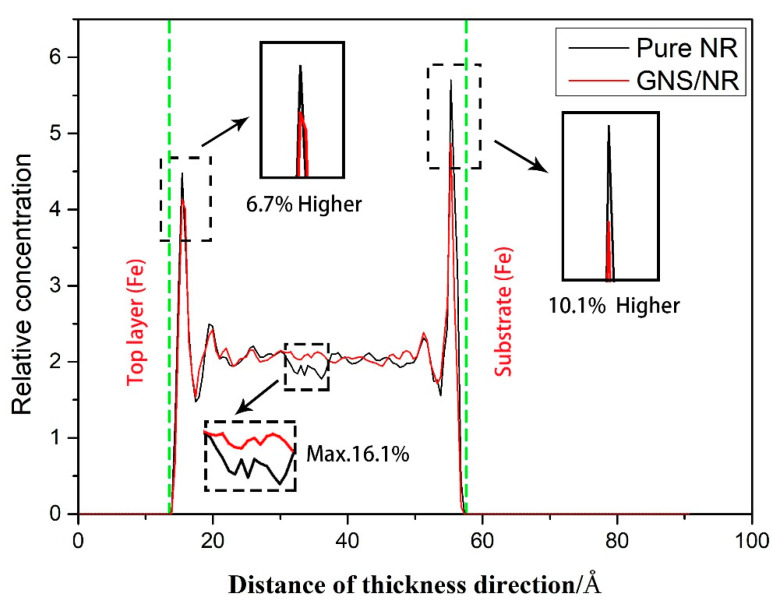
Relative atomic concentration distribution along the thickness direction of the pure NR and GNS/NR composites.

To further reveal the microscopic wear mechanism of the tribological properties of GNS-reinforced NR polymer composites, the atomic relative concentration distribution profiles along the thickness direction during the friction process were calculated for both materials, as shown in Figure 10.

From Figure 10, the peaks of the relative atomic concentrations of both materials can be observed at 14 Å (top contact region) and 57 Å (bottom contact region) along the material matrix thickness direction, and the relative atomic concentrations of the pure NR composite matrix were 6.7% and 10.1% higher than those of the GNS/NR matrix near both locations, respectively. At the same time, the average relative atomic concentration of the GNS/NR matrix inside the substrate (at about 26 Å along the thickness direction) was 16.1% higher than that of the NR matrix. For the GNS/NR composite, the atoms inside the matrix were adsorbed around the GNS surface; thus, the metal Fe atomic interaction between the upper and lower layers was weakened, and the anti-friction capability was improved.

Due to VDW forces and adsorption effects [25], an interaction interface is formed between graphene and the polymer during the frictional wear process. Therefore, the interaction energy between graphene and the natural rubber matrix during the friction process is calculated and simulated based on Equation (9).

(9) $$E_{inter}=E_{total}-E_{NR}-E_{GNS}$$

**Figure 11:**
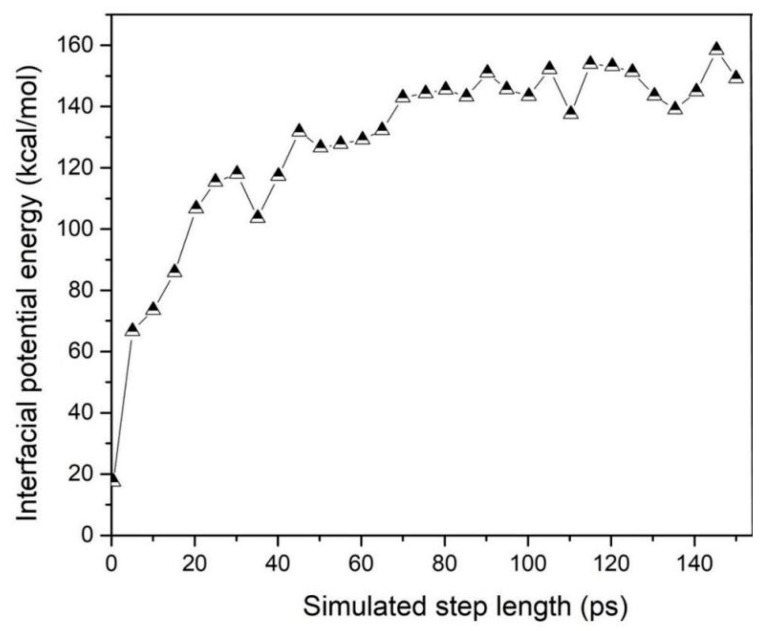
Graph of potential energy of GNS-NR interaction with simulation time.

The frictional wear mechanism was analyzed from the perspective of energy change during material friction, and the change in the interaction potential energy between graphene and natural rubber was extracted by the frictional trajectory file; and the results are shown in Figure 11. The mutual potential energy is 17.5 kcal/mol and 145.5 kcal/mol, respectively, at the initial state (0 ps), reaching dynamic equilibrium (80 ps), and its potential energy change value increases more than 8 times, indicating the increase in interfacial interaction potential energy during frictional wear. It shows that there is a strong affinity between the GNS and NR molecular chains [26,27]. The strong interaction between the filler and the rubber leads to an enhanced adsorption between the graphene and the natural rubber matrix. The effective adsorption of the natural rubber molecular chains around the surface, which in turn leads to the reduction of natural rubber molecular chains present at the frictional interface location, reduces the interaction between the polymer matrix and the metal layer and improves the wear resistance of the natural rubber matrix.

## 4. Conclusions

Two microscopic scale molecular dynamics models pure NR and GNS/NR were developed and simulated to compare the thermodynamic and tribological properties of the two material models.

Simulations of molecular dynamics have shown that the addition of graphene significantly increases the elastic modulus, bulk modulus, and shear modulus. Significant improvements have been made to the material’s tensile characteristics, resistance to deformation, and shear resistance. The enhanced orientation of the molecular chains and the ordered structure result in a weakening of the mean free range of phonon diffusion, an increase in phonon scattering, and an increase in thermal conductivity. The stiffness of the molecular chains increases, but their flexibility and resistance to low temperatures decrease.

The analysis of the microscopic information from the friction interface revealed that the atomic velocity at the friction interface between the Pure NR matrix and the metal Fe layer was faster, the atomic concentration was higher, the temperature increase at the friction interface was greater, and the system energy was higher. The addition of GNS significantly improved all the action factors at the friction interface of the composite matrix, demonstrating the enhancement of the tribological properties of the NR material matrix by GNS. This study provides a new method and theoretical basis for predicting the thermomechanical and tribological properties of inorganic nanomaterial-reinforced polymer composites.

## Figures and Tables

**Table 1:** Mechanical properties of the pure natural rubber and the graphene/natural rubber composites.

Samples	Shear Modulus/GPa	Increase (%)	Bulk Modulus/GPa	Increase (%)
Pure NR	1.441	0	1.150	0
GNS/NR	2.807	94.8	2.416	110.1

**Table 2:** Thermal conductivity of the pure natural rubber and the graphene/natural rubber composites.

Samples	Pure NR	GNS/NR
Thermal conductivity (W·m^−1^·K^−1^)	0.1482	0.2355

## Data Availability

The data presented in this study are available upon request from the corresponding author.
